# The Angiotensin Converting Enzyme Deletion/Deletion Genotype Is a Risk Factor for Severe COVID-19: Implication and Utility for Patients Admitted to Emergency Department

**DOI:** 10.3390/medicina57080844

**Published:** 2021-08-20

**Authors:** Anna Annunziata, Antonietta Coppola, Valentina Di Spirito, Rosa Cauteruccio, Antonella Marotta, Pierpaolo Di Micco, Giuseppe Fiorentino

**Affiliations:** 1 Sub-Intensive Care Unit, Department of Respiratory Pathophysiology and Rehabilitation Monaldi—A.O. Dei Colli, 80131 Naples, Italy; anna.annunziata@gmail.com (A.A.); valentina.dispirito@gmail.com (V.D.S.); rosa.cauteruccio@gmail.com (R.C.); antonella.marotta@gmail.com (A.M.); giuseppe.fiorentino1@gmail.com (G.F.); 2 Department of Medicine, Ospedale Buonconsiglio Fatebenefratelli di Napoli, 80131 Naples, Italy; pdimicco@libero.it

**Keywords:** angiotensin converting enzyme, COVID-19, ARDS

## Abstract

*Background and objective*: Insertion/deletion polymorphisms of angiotensin-converting enzyme (ACE) have been previously described in association with adult respiratory distress syndrome (ARDS) and correlated to outcome. The ACE deletion/deletion(D/D)genotype represents a marker of thrombosis in subjects apparently without predisposing factors and/or traditional thrombophilic alterations and increases the risk of venous thromboembolism in subjects in whom a thrombogenic condition occurs. Thrombosis seems to play a role very early in the disease caused by SARS-CoV-2, in particular in those with severe COVID-19 pneumonia. The counterbalance between angiotensin-converting enzyme (ACE) and ACE2 activities in COVID-19 disease may play a crucial role in the thrombo-inflammatory process. We hypothesised that a genetic predisposition could condition the severity and complications of SARS-CoV-2 infection. *Materials and methods*: We conducted a spontaneous, single centre observational study in the Sub-Intensive Care Unit of A.O.R.N. Ospedali dei Colli, Cotugno Hospital, Naples (Italy). In this study, we performed genetic screening for ACE D/D genotype and other thrombophilic mutations in 20 patients affected by ARDS related to COVID-19 pneumonia, compared to 19 age- and sex-matched healthy controls. *Results*: All tested patients had multiple polymorphisms and, in particular, a significantly higher prevalence of ACE D/D polymorphism in severe COVID-19 patients *Conclusion*: We found that the majority of patients who tested positive for ACE D-D genotype and who were not associated with other risk factors for VTE showed an evolution to ARDS. This finding could have a predicting role in the selection of patients more prone to developing severe COVID-19 during clinical observation in emergency department.

## 1. Introduction

Insertion (I)/deletion (D) polymorphisms of angiotensin-converting enzyme (ACE) have been previously described in association with adult respiratory distress syndrome (ARDS) and correlated with its outcome [1]. Pneumonia secondary to SARS-CoV-2 infection, named COVID-19, is associated with an increase in the permeability of the alveolar-capillary barrier as reported in several reports, leading to ARDS [2]. ACE is a metallopeptidase that converts angiotensin I to angiotensin II (Ang II), which acts as a vasoconstrictor, also degrading bradykinin that may interact with the clotting system. The SARS-CoV-2 spike protein enters host cells via the angiotensin-converting enzyme 2 (ACE2) receptor on the surface of pulmonary type 2 alveolar cells [3]. ACE-2 physiologically converts AngII to angiotensin 1–7, and it is a negative regulator of the system. From a clinical point of view, in a small cohort study, levels of Ang II were found to be markedly increased in COVID-19 plasma samples [4]. Further studies have reported high Ang II levels in mice infected with SARS-CoV-2 and showed that Ang II levels were related to the severity of disease, while mice deficient for ACE showed markedly improved disease [5]. Several studies have also demonstrated an association between the frequency of ACE deletion/deletion (D/D) polymorphisms and both the prevalence and the mortality rates of COVID-19 disease [5,6].

Based on these findings, the counterbalance between ACE and ACE2 activities in COVID-19 may play a crucial role in the thrombo-inflammatory processes described in severe pneumonia with evolution in ARDS, and the D/D genotype of ACE could also be a part of this crucial pathophysiological aspect [7,8,9,10,11].

In this study, our goal was to understand if there could be a genetic predisposition in development of critical COVID-19 and its complications. We tested thrombophilic mutations for inherited thrombophilia in patients admitted to the emergency department for COVID-19 and compared them with a control group. We observed that the ACE DD genotype appears to be associated with a more severe course of COVID-19 compared to the other thrombophilic polymorphisms that we tested. Thus, we could suggest fast testing of the ACE I/D polymorphism for patients admitted to the emergency department in order to pre-select patients prone to developing a more aggressive clinical disease.

## 2. Material and Methods

We enrolled 24 patients, 17 male and 7 female, aged 57.5 ± 13.5 years. Patients were affected by severe COVID-19 and admitted to our intensive care unit between 16 March 2020 and 1 April 2020. We obtained consent from 20 patients. The control population included 19 healthy subjects, comparable in terms of sex and age, randomly recruited from the staff of the hospital and from blood donors of the same ethnicity (Caucasian) and geographical area (southern Italy) as the patients.

The diagnosis of COVID-19 was made according to the World Health Organisation interim guidance and confirmed by RNA detection of SARS-CoV-2 in a nasopharyngeal swab. Severe COVID-19 was defined as meeting any one of the following criteria: respiratory rate ≥30 breaths/min; arterial oxygen saturation ≤93% at rest; PaO_2_/FiO_2_ ≤ 300 mmHg. At admission, all patients had severe respiratory failure with PaO_2_/FiO_2_ < 150 mmHg and underwent mechanical ventilation.

The baseline characteristics of the patients are described in Table 1; 11 of them (55%) were affected by hypertension, and the average body mass index (BMI) was 27.6 ± 2.3; 14 (70%) developed thrombosis or acute pulmonary embolism, although all patients underwent prophylaxis with low molecular weight heparin at admission. We hypothesised that a genetic predisposition affects the severity and complications of SARS-CoV-2 infection.

We performed genetic tests, determined by the real time polymerase chain reaction amplification method for angiotensin-converting enzyme (ACE) I/D. We also tested for thrombophilia-associated polymorphisms, including factor V Leiden (FVL), factor V H1299 R (factor V HR2), plasminogen activator inhibitor-1675 4G/5G (PAI-1675), methylene tetrahydrofolate reductase (MTHFR) C677T, and MTHFR A1298C.

## 3. Results

Twenty patients were subjected to genetic tests, and four patients did not give consent. We observed that 17 patients presented the ACE D/D genotype, 2 were I/D, and 1 was I/I. Moreover, 19 healthy subjects were enrolled as a control group.

We noticed that all tested patients had multiple polymorphisms and, in particular, a significantly higher prevalence of ACE D/D polymorphism in severe COVID-19 patients. These elements induce a hypercoagulable state and increase the risk of thromboembolic events. We found that 60% of patients had a mutation for MTHFR C677 T, 60% had a mutation for MTHFR A1298C, and 85% had mutation for plasminogen activator inhibitor-1 675 4G/5G (Table 2). In summary, we found that 85% of our critically ill patients had the ACE D/D, the ACE I/D genotype was found in 10%, and 100% had multiple heterozygosity and homozygosity for factors related to inherited thrombophilia. Compared to healthy subjects, there was a statistically significant difference in critically ill COVID-19 patients. COVID-19 patients more frequently had FV Leiden heterozygosity (*p* = 0.046), the ACE D/D genotype (*p* = 0.022), and the PAI 4G/4G genotype (*p* = 0.008) In the group of our 19 healthy controls, no patients with V Leiden mutations were identified (*p* = 0.047), 8 out of 19 showed the ACE I/D genotype (*p* = 0.008), 16 out of 19 controls showed the PAI-1-675 4G/5G genotype (*p* = 0.008), and no statistically significant differences were found for the other gene (Table 2).

## 4. Discussion

The clinical course of COVID-19 is characterized by a bilateral interstitial pneumonia that may lead to lung failure and other severe consequences till death; yet, several troubles in the daily clinical management of COVID-19 have also been related to a prolonged duration of hospitalization for the majority of inpatients because the clinical scenario and lung performance may change during the clinical course of the disease. Thus, several tests have been suggested to select patients that are more prone to develop a more aggressive clinical form of COVID-19, and because the link between SARS-CoV-2 and the ACE2 receptor is well known, ACE I/D has also been suggested as a potential useful test to select patients more at risk of severe COVID-19 early on [9,12,13]. On the other hand, different studies have associated the ACE D/D genotype with an increased risk of cardiovascular pathologies, due to a consequent increase in plasma levels of ACE (twofold higher compared to subjects with genotype II) [14]. Some authors have also shown that the ACE D/D genotype represents a susceptibility marker for thrombosis in subjects apparently without predisposing factors and traditional thrombophilic alterations and increases the risk of venous thromboembolism in subjects in whom a thrombogenic condition occurs [15,16]. Furthermore, an association between an ACE I/D polymorphism (the D allele of the human ACE gene confers increased ACE activity in plasma), and pulmonary hypertension has been reported; this association is, however, controversial. For instance, one study showed that the ACE D/D genotype is associated with less right ventricular hypertrophy [10], whereas another reported a correlation between the ACE D/D genotype and the severity of symptoms [11]. This pathophysiological background could also influence the trend to VTE that several inpatients with COVID-19 showed.

During pandemic, there has been association between inherited thrombophilia and severe COVID-19 because its association with VT has been established with non-univocal data [12].

Of course, in our clinical report, together with the ACE D/D genotype, other common thrombophilic gene polymorphisms have also been investigated.

Although, in the literature, there are few studies regarding genetic factors in severe COVID-19 patients, particular attention should be given to several polymorphisms, such as factor V Leiden and prothrombin A20210G, because of their independent association with recurrent VTE [13,14,15].

Intriguingly, a relevant role in predicting severe COVID-19 in our population has also been found regarding the ACE D-D genotype; from a pathophysiological point of view, this association could also be related to the involvement of ACE in the pathological mechanism of SARS-CoV-2 in the respiratory tract [16]. Furthermore, this finding could also have a predicting role in the selection of patients more prone to develop severe COVID-19 during clinical observation in the emergency department. Thus, further studies, on larger populations, are required to assess the clinical outcome of COVID-19 infection in ACE D/D, I/D, and I/I patients and to study the exact role of ACE polymorphisms in COVID-19 disease regarding the evolution to ARDS and to VTE.

In this way, we found that the majority of patients who tested positive for the ACE D-D genotype and who were not associated with other risk factors for VTE showed an evolution to ARDS. The limit of the study is represented by the small sample examined. Although this observation could be only of indicative value because of the relatively small sample size analysed, because the data in the literature are lacking in this scenario, we believe that the clinical utility of these data per se is concrete and that they could also be useful as preliminary screening in emergency departments to select potentially frail patients for lung failure.

## Figures and Tables

**Table 1:** Baseline characteristics of COVID-19 patients undergoing genetic tests.

Age (mean ± DS)	57.5 ± 13.5
Male sex *n* (%)	14/20 (70%)
BMI (mean ± DS)	27.6 ± 2.3
Hypertension *n* (%)	11/20 (55%)
Diabetes *n* (%)	3/20 (15%)
CAD *n* (%)	2/20 (10%)
COPD *n* (%)	3/20 (15%)
Cancer *n* (%)	1/20 (5%)
Charlson Index	
0–1	8/20 (40%)
2–3	6/20 (30%)
4–5	6/20 (30%)
paO_2_/FiO_2_ (mean ± DS)	158.9 ± 94.9
Mortality *n* (%)	1 (5%)
Pulmonary Embolism *n* (%)	14/20 (70%)

Legend to Table 1: BMI, body mass index; CAD, coronary heart disease; COPD, chronic obstructive pulmonary disease; DS: standard deviation.

**Table 2:** Number of critically ill COVID-19 patients for each test result.

Test Performed	Severe COVID-19 Patients	Healthy Subjects	*p* Value
F V Leiden			
Wild Tipe	15	19	0.047
Heterozygous	5	0	0.046
Homozygous	0	0	NS
F V H2R			
Wild Tipe	15	17	0.656
Heterozygous	5	2	0.407
Homozygous	0	0	NS
MTHFR C677T			
Wild Tipe	8	3	0.155
Heterozygous	6	10	0.200
Homozygous	6	6	1.000
MTHFR A1298C			
Wild Tipe	8	11	0.527
Heterozygous	6	7	1.000
Homozygous	6	1	0.091
PAI-1-675			
5G/5G	3	2	1.000
4G/5G	8	16	0.008
4G/4G	9	1	0.008
ACE			
ACE I/I	1	3	0.341
ACE I/D	2	8	0.008
ACE D/D	17	8	0.022

Legend to Table 2: F V Leiden: factor V Leiden; F V H2R: factor VH2R variant; MTHFR C677T: methylentetrahydrofolate reductase C677T variant; MTHFR A1298C: methylentetrahydrofolate reductase A1298C variant; PAI-1-675: plasminogen activator inhibitor-1675; ACE: angiotensin-converting enzyme; NS: not significant.

## Data Availability

Data supporting the reported results can be found in the dataset placed c/o Ospedale Monaldi, Naples.
